# Host–Microbiota Interactions in Adolescents with Severe Anorexia Nervosa: Linking Gut Dysbiosis, Intestinal Barrier Function and Microbial Metabolism

**DOI:** 10.3390/nu18193136

**Published:** 2026-09-23

**Authors:** Livia Gargiullo, Federica Del Chierico, Maria Rosaria Marchili, Mariangela Irrera, Italo Pretelli, Matteo Scanu, Francesca Toto, Annalisa Grandin, Rosaria Marotta, Cristina Mascolo, Flavia Cirillo, Valentina Colistra, Ludovica Ricci, Valeria Zanna, Lorenza Putignani, Alberto Villani

**Affiliations:** 1 General Pediatrics, Infectious Diseases and Level II Pediatric Emergency Department Unit, Bambino Gesù Children’s Hospital, IRCCS, 00165 Rome, Italy; mrosaria.marchili@opbg.net (M.R.M.); mariangela.irrera@opbg.net (M.I.); annalisa.grandin@opbg.net (A.G.); rosaria.marotta@opbg.net (R.M.); cristina.mascolo@opbg.net (C.M.); flavia.cirillo@opbg.net (F.C.); valentina.colistra@opbg.net (V.C.); alberto.villani@opbg.net (A.V.); 2 Unit of Microbiome, Bambino Gesù Children’s Hospital, IRCCS, 00146 Rome, Italy; federica.delchierico@opbg.net (F.D.C.); matteo.scanu@opbg.net (M.S.); francesca.toto@opbg.net (F.T.); 3 Anorexia and Eating Disorders Unit, Child and Adolescent Psychiatry, Bambino Gesù Children’s Hospital, IRCCS, 00165 Rome, Italy; italo.pretelli@opbg.net (I.P.); valeria.zanna@opbg.net (V.Z.); 4 Pediatrics Residency Program, Department of Pediatrics, University of Rome Tor Vergata, 00133 Rome, Italy; ludovica.ricci@opbg.net; 5 Department of Life Sciences, Health and Health Professions, Link Campus University, 00165 Rome, Italy; lorenza.putignani@opbg.net; 6 Unit of Microbiomics and Unit of Microbiome, Bambino Gesù Children’s Hospital, IRCCS, 00146 Rome, Italy; 7 Systems Medicine Departments, University of Rome Tor Vergata, 00133 Rome, Italy

**Keywords:** anorexia nervosa, gut microbiota, gut–brain axis, biomarkers, intestinal permeability, nutritional rehabilitation

## Abstract

Background: Anorexia nervosa (AN) is increasingly recognized as a multisystem disorder involving complex host–microbiota interactions, yet prospective pediatric studies with a comprehensive, longitudinal characterization of host–microbiota interactions remain scarce. Methods: In this prospective longitudinal study, 26 adolescents with severe AN were evaluated at hospital admission (T0), after one month (T1), and after three months of inpatient nutritional rehabilitation (T2). Gut microbiota composition was assessed by 16S rRNA sequencing and compared with healthy controls (HC) at the baseline; predicted functional profiling was performed using PICRUSt2. Serum and urinary biomarkers of intestinal barrier integrity and microbial metabolism, routine clinical and biochemical parameters, and psychometric scores (CDI-2, MASC-2) were evaluated longitudinally using linear mixed-effects models. Results: At the baseline, adolescents with AN showed reduced bacterial richness compared with healthy controls, with significantly lower Chao1 diversity (*p* = 0.00013), whereas the Shannon and Simpson indices did not differ significantly (*p* = 0.54 and *p* = 0.27, respectively). Microbial community composition differed significantly between groups based on Bray–Curtis (R^2^ = 0.0724, FDR-adjusted *p* = 0.001) and unweighted UniFrac (R^2^ = 0.0540, FDR-adjusted *p* = 0.001) distances. LEfSe identified 19 differentially abundant taxa; among the strongest discriminators, *Blautia*_A_141781, *Romboutsia*_B, and *Clostridium*_T were enriched in AN, whereas *Akkermansia*, *Phocaeicola*_A_858004, and *Bacteroides*_H were enriched in the healthy controls. Nutritional rehabilitation was associated with partial microbiota remodeling, alongside significant improvements in BMI and heart rate. Several host-derived biomarkers, including urinary indican, serotonin, nitric oxide, and serum occludin, changed significantly over time, whereas bile acids, short-chain fatty acids, IgA, lipopolysaccharide, and haptoglobin remained stable. Conclusions: Gut microbiota and host-derived biomarkers evolve dynamically during nutritional rehabilitation in adolescents with severe AN, supporting their potential role as complementary tools for monitoring biological recovery.

## 1. Introduction

Anorexia nervosa (AN) is a severe psychiatric disorder characterized by persistent energy restriction, intense fear of weight gain, and a distorted perception of body image, resulting in marked weight loss and profound metabolic alterations [1]. Although traditionally considered a primary psychiatric illness, AN is now recognized as a complex multisystem disorder involving endocrine, metabolic, immunological, and gastrointestinal dysfunctions that contribute to disease onset, progression, and persistence [2,3,4]. Adolescence represents the period of highest disease incidence, and prolonged malnutrition during this critical stage of development profoundly affects physical growth, neurodevelopment, immune function, and psychosocial well-being [2]. Despite advances in multidisciplinary management, AN remains associated with the highest mortality rate among psychiatric disorders and a high risk of relapse, highlighting the need to better understand the biological mechanisms underlying its pathophysiology [2].

In recent years, increasing evidence has identified the gut microbiota as a key component of the microbiota–gut–brain axis, a bidirectional communication network linking the gastrointestinal tract with the central nervous system through neural, endocrine, metabolic, and immune pathways. Beyond its fundamental role in nutrient metabolism, the gut microbiota contributes to the regulation of intestinal barrier integrity, immune homeostasis, and the production of numerous bioactive compounds, including short-chain fatty acids (SCFAs), bile acids, tryptophan metabolites, and neurotransmitters such as serotonin, dopamine, and γ-aminobutyric acid (GABA), all of which may influence appetite regulation, mood, cognition, and eating behavior [5,6,7].

Several lines of evidence indicate how these pathways may operate. In preclinical models, germ-free and antibiotic-treated rodents display altered anxiety-like behavior and exaggerated hypothalamic–pituitary–adrenal axis reactivity that can be normalized by microbial colonization, and germ-free mice receiving fecal microbiota from patients with AN have been reported to show reduced food intake, impaired weight gain and increased anxiety-related behavior [3]. Mechanistically, gut bacteria regulate the availability of tryptophan for host serotonin synthesis and stimulate enteric serotonin production by enterochromaffin cells, modulate vagal afferent signaling and central gene expression through SCFAs and other fermentation products, and generate bacterial proteins such as caseinolytic protease B (ClpB), which shares sequence homology with α-melanocyte-stimulating hormone and may interfere with the satiety and anxiety pathways [2,6]. In clinical cohorts, lower microbial richness has been associated with higher depressive symptom and eating-disorder psychopathology scores in patients with AN, and changes in specific taxa during refeeding have been correlated with changes in mood and gastrointestinal symptoms, although the findings are heterogeneous and largely derived from small adult samples [6].

Accordingly, numerous studies have demonstrated that patients with AN harbor an altered gut microbial ecosystem compared with healthy individuals. Although the specific microbial signatures vary among cohorts, dysbiosis is consistently characterized by alterations in microbial diversity, depletion of beneficial SCFA-producing bacteria, enrichment of taxa adapted to nutrient deprivation, and functional changes involving microbial metabolic pathways [8,9,10]. Notably, the large multi-omics study by Fan et al. combined shotgun metagenomics, serum metabolomics, and fecal microbiota transplantation in germ-free mice, providing mechanistic evidence that gut microbial alterations may actively contribute to AN pathogenesis rather than simply represent a consequence of starvation [9].

Beyond taxonomic alterations, growing evidence suggests that intestinal dysfunction in AN also involves impaired epithelial barrier integrity, altered mucosal immunity, bacterial translocation, and microbial metabolic dysregulation. Increased intestinal permeability may facilitate the systemic translocation of bacterial products such as lipopolysaccharide (LPS), promoting immune activation and low- to high-grade inflammation, while altered microbial metabolism may affect the production of SCFAs, bile acids, and neurotransmitters involved in gut–brain communication [5,6,7]. These biological alterations may contribute not only to the metabolic consequences of malnutrition but also to the maintenance of gastrointestinal symptoms and psychiatric manifestations frequently observed in patients with AN.

Longitudinal studies have further shown that gut dysbiosis may persist despite improvements in body weight during hospitalization, suggesting that microbial restoration does not necessarily parallel short-term clinical improvement. Persistent alterations in gut microbial composition and function have been observed following inpatient treatment, indicating that microbiota recovery may require longer periods or additional targeted interventions [4,11]. However, although previous studies have considerably expanded our understanding of gut microbial alterations in AN, three specific gaps remain. First, most cohorts have included adult women, whereas adolescents with severe AN, in whom malnutrition occurs during a critical window of microbial and neurodevelopmental maturation, are markedly under-represented. Second, most available reports are cross-sectional or include only two sampling points, so the trajectory of microbial change during the early phase of inpatient refeeding has rarely been described. Third, microbiota profiling has generally been performed in isolation, without concurrent measurement of intestinal barrier integrity, mucosal immunity and microbial metabolic activity in the same patients, and without linkage to routine clinical and biochemical parameters or to validated psychometric instruments. It therefore remains unknown whether host-derived biomarkers of barrier function and microbial metabolism change in parallel with microbiota remodeling during nutritional rehabilitation in adolescents.

Therefore, this prospective longitudinal study investigated gut microbiota trajectories in adolescents with severe anorexia nervosa, from hospital admission through the first three months of inpatient treatment. By integrating longitudinal microbiota profiling with clinical, biochemical and psychometric assessments, together with biomarkers of intestinal barrier function, immune activation and microbial metabolism, we aimed to provide a comprehensive characterization of host–microbiota interactions and to explore their relationships with disease severity.

We hypothesized that (i) at hospital admission, adolescents with severe AN would show reduced bacterial richness and a distinct taxonomic and predicted functional profile compared with healthy controls; (ii) three months of nutritional rehabilitation would be accompanied by partial rather than complete remodeling of the gut microbial community; and (iii) host-derived biomarkers of intestinal barrier integrity and microbial metabolism would change measurably over the same period and would be associated with clinical, biochemical and psychometric indices of recovery. The first hypothesis was addressed by a pre-specified comparison with healthy controls, whereas the analyses addressing the second and third hypotheses were exploratory.

## 2. Materials and Methods

### 2.1. Study Design and Setting

This prospective observational study was conducted at the General Pediatrics, Infectious Diseases and Level II Pediatric Emergency Department Unit and the Anorexia and Eating Disorders Unit, Child and Adolescent Psychiatry Unit of Bambino Gesù Children’s Hospital, IRCCS (Rome, Italy), between 10 September 2025 and 30 March 2026.

### 2.2. Participants and Eligibility Criteria

Adolescents consecutively admitted with a diagnosis of severe anorexia nervosa (AN) according to the Diagnostic and Statistical Manual of Mental Disorders, Fifth Edition, Text Revision (DSM-5-TR) [1] were invited to participate.

Inclusion criteria were: (i) age between 11 and <18 years; (ii) a diagnosis of AN with a body mass index (BMI) ≤ 16 kg/m^2^ at admission, adopted as an operational enrolment threshold; since DSM-5-TR bases the severity specifier on absolute BMI only in adults and indicates that the corresponding BMI percentiles should be used in children and adolescents [1], nutritional status and disease severity were additionally characterized in age- and sex-specific terms, as detailed in Section 2.5; and (iii) written informed consent from patients and/or their parents or legal guardians.

Exclusion criteria, pre-specified in the study protocol, were: (i) food intolerances or any other ongoing metabolic or gastrointestinal disease; (ii) intellectual disability or any other neurodevelopmental disorder; and (iii) refusal of the patient and/or of the parents or legal guardians to take part in the study. The criteria used to select healthy controls are detailed in Section 2.4.

### 2.3. Nutritional Rehabilitation Protocol and Follow-Up

Nutritional rehabilitation followed a standardized, multidisciplinary institutional protocol for the inpatient management of adolescents with severe AN, whose safety and efficacy have previously been validated in a five-year cohort of hospitalized patients at our center [12]. Briefly, the nutritional plan was individualized according to the proportion of the estimated caloric requirement spontaneously tolerated by oral intake at admission, integrating oral feeding, oral nutritional supplements, and when indicated, nasogastric enteral feeding, with caloric intake progressively increased according to weight trend under close multidisciplinary monitoring of fluid balance, electrolytes, and vital parameters to minimize the risk of refeeding syndrome. The feeding route actually delivered to each patient during follow-up (exclusive oral intake, oral nutritional supplements or supplementary nasogastric enteral feeding) was not systematically recorded and could therefore not be analyzed as a covariate. No predefined weight target was set as a study outcome: caloric intake was progressively adjusted according to the individual weight trend, and body weight and BMI were analyzed as continuous variables over time.

Clinical evaluations and biological samples were collected at hospital admission (T0), after one month (T_1_, ±15 days), and after three months of inpatient treatment (T2, ±15 days), whenever available according to the clinical course and patient follow-up.

Missing follow-up data (due to transfer to other healthcare facilities or failure to attend scheduled visits) were handled using linear mixed-effects models, which allowed for the inclusion of all available observations without excluding participants with incomplete follow-up.

The planned sample size was approximately 30 patients, as prospectively defined in the study protocol based on the expected hospital caseload. Given the observational and descriptive nature of the study, no formal a priori power calculation was performed. The primary objective was to characterize gut microbiota dysbiosis at hospital admission, whereas longitudinal and association analyses were considered exploratory.

### 2.4. Healthy Controls

Fecal samples from healthy subjects (healthy controls, HC) included in the present study were obtained from the reference Microbiome Special Collection of the OPBG BBMRI biobank, established through an epidemiological survey conducted at the Microbiome Research Unit of Bambino Gesù Children’s Hospital, IRCCS, EUBIOM Project, to generate a reference biobank of samples from healthy subjects.

For HC subjects, we considered the following inclusion criteria: age between 11 and <18 years; (ii) eating style that follows a Mediterranean diet; and (iii) written informed consent from patients and/or their parents or legal guardians. The following exclusion criteria were considered for all subjects: history of chronic inflammatory diseases of the gastrointestinal (GI) tract; history of allergies; intake of antibiotics and/or pre/probiotics in the last 3 months; history of gastroenteritis and/or parasitosis in the last 3 months; particular dietary regimes (vegetarian, vegan diet, etc.); a family history of chronic or autoimmune diseases, BMI ≤ 18.4 or BMI > 24.9; history of other neurological, gastrointestinal, allergic, and psychiatric pathologies.

The biobank comprises biological samples collected from HC pediatric subjects and was approved by the Ethics Committee of Bambino Gesù Children’s Hospital (Protocol No. 2839_OPBG_2022; Approval No. 1076, 26 July 2022; CE Version 1.0, 05/09/2022 EUBIOM/2839_OPBG_2022).

### 2.5. Clinical and Psychological Assessment

Anthropometric, cardiovascular, laboratory, and psychological data were collected from the electronic medical records at each study visit.

Anthropometric measurements included body mass index (BMI, kg/m^2^), BMI-for-age percentile and BMI z-score, obtained from the 2000 CDC growth reference for age and sex [13], and the percentage of median BMI (%mBMI), calculated as the ratio between the measured BMI and the CDC 50th percentile BMI for age and sex. The degree of malnutrition was graded as mild (%mBMI 80–90), moderate (70–79) or severe (<70) according to the position paper of the Society for Adolescent Health and Medicine [14]. Because BMI-based severity specifiers have been shown to have limited clinical validity in children and adolescents [15], disease severity was additionally anchored to the clinical criteria that determined inpatient admission, in line with the DSM-5-TR indication that the level of severity may be increased to reflect clinical symptoms, the degree of functional disability, and the need for supervision [1]. Cardiovascular assessment comprised systolic and diastolic blood pressure and heart rate.

Routine laboratory investigations included white blood cell count, neutrophil count, lymphocyte count, hemoglobin, platelet count, creatinine, blood urea nitrogen, aspartate aminotransferase (AST), alanine aminotransferase (ALT), glucose, sodium, phosphorus, magnesium, total calcium, vitamin C, vitamin D, ferritin, and folate concentrations.

Psychological assessment was performed as part of the routine multidisciplinary clinical evaluation using the Children’s Depression Inventory, Second Edition, Self-Report (CDI-2; Kovacs M. Children’s Depression Inventory 2 (CDI-2): Technical Manual. Multi-Health Systems; 2011) and the Multidimensional Anxiety Scale for Children, Second Edition, Self-Report (MASC-2; March JS. Multidimensional Anxiety Scale for Children, Second Edition (MASC-2): Technical Manual. Multi-Health Systems; 2013).

The CDI-2 is a validated self-report questionnaire designed to assess depressive symptoms in children and adolescents, whereas the MASC-2 evaluates multiple domains of anxiety. For both instruments, standardized T-scores were used for statistical analyses. According to the respective administration manuals, T-scores were classified as <40 (low), 40–59 (within the normal range), 60–64 (mildly elevated), 65–69 (clinically significant), and ≥70 (severely clinically significant). Both continuous T-scores and severity categories were included in the analyses.

### 2.6. Collection of Biological Samples and Host Biomarker Assessment

Fecal, blood, and urine samples were collected at each study visit according to standardized clinical procedures. Blood and urine samples were obtained in the morning in the fasting state, as part of the periodic routine biochemical monitoring, and were transferred fresh to the hospital laboratory. Fecal samples were passed spontaneously and delivered fresh to the laboratory within two hours whenever possible; otherwise they were stored at +4 °C until transfer. Blood samples were centrifuged to obtain serum, whereas fecal and urine samples were processed immediately after collection. All specimens were aliquoted and stored at −80 °C until analysis at the Microbiome Research Unit, Bambino Gesù Children’s Hospital, IRCCS (Rome, Italy).

To characterize host–microbiota interactions, serum and urinary biomarkers representing complementary biological pathways were quantified. Biomarkers of intestinal barrier integrity and bacterial translocation included serum occludin and lipopolysaccharide (LPS). Mucosal immune activation and systemic inflammation were assessed by serum immunoglobulin A (IgA) and haptoglobin (Hp), respectively. Microbial metabolic activity was evaluated through serum SCFAs, whereas urinary indican and bile acids were measured as indicators of intestinal dysbiosis and gut microbiota-mediated bile acid metabolism. Finally, urinary serotonin and nitric oxide were quantified as surrogate markers of gut–brain and gastrointestinal signaling.

Serum LPS concentrations were measured using a commercial ELISA kit (Hycult Biotechnology, Uden, The Netherlands; assay range 0.04–10.0 EU/mL), with absorbance recorded at 405 nm.

Serum occludin and IgA were quantified using commercial ELISA kits (Fine Biotech Co., Wuhan, China; assay ranges: 0.156–10 ng/mL and 25–200 ng/mL, respectively), with absorbance measured at 450 nm.

Serum haptoglobin was determined using a Human Haptoglobin ELISA Kit (Bioassay Technology Laboratory, Shanghai, China; assay range 2–600 μg/mL), whereas serum SCFAs were quantified using a Human SCFA ELISA Kit (AFG Bioscience LLC, Northbrook, IL, USA; assay range 1.5–48 μmol/L). Both assays were read at 450 nm.

Urinary indican was measured using the Quantichrom™ Indican Assay Kit (BioAssay Systems, Hayward, CA, USA; assay range 0.2–20 mg/dL) at 480 nm. Urinary nitric oxide was quantified using the Nitric Oxide Assay Kit (Sigma-Aldrich, St. Louis, MO, USA; assay range 0.6–200 μM) at 540 nm. Urinary serotonin was measured using a competitive ELISA kit (Thermo Fisher Scientific, Waltham, MA, USA; assay range 15.63–1000 ng/mL) at 450 nm. Urinary bile acids were quantified using the Bile Acid Assay Kit (Sigma-Aldrich, St. Louis, MO, USA; linear range 1–150 μM), with fluorescence measured at excitation/emission wavelengths of 530/585 nm.

### 2.7. DNA Extraction, 16S rRNA Gene Sequencing and Bioinformatic Processing

Genomic DNA was extracted from approximately 200 mg of fecal material using the QIAamp Fast DNA Stool Mini Kit (Qiagen, Hilden, Germany), according to the manufacturer’s instructions.

The bacterial community was profiled by amplification of the V3–V4 hypervariable region (~460 bp) of the 16S rRNA gene using the primer pair recommended in the Illumina MiSeq 16S Metagenomic Sequencing Library Preparation protocol (Illumina, San Diego, CA, USA). Amplicons were purified and indexed using the Nextera XT Index Kit (Illumina, San Diego, CA, USA). Library concentrations were determined with the Quant-iT™ PicoGreen^®^ dsDNA Assay Kit (Thermo Fisher Scientific, Waltham, MA, USA), normalized to equimolar concentrations, pooled, and sequenced on an Illumina MiSeq platform using paired-end chemistry (2 × 300 bp), according to the manufacturer’s instructions.

Positive and negative controls were included throughout DNA extraction, library preparation, and sequencing to monitor potential contamination. No amplification was detected in the negative controls; therefore, no contaminant ASVs were identified or removed prior to downstream analyses.

Raw sequencing reads (FASTQ files) were imported into QIIME2 (version 2024.5). Sequence denoising, quality filtering, paired-end merging, and chimera removal were performed using the DADA2 plugin to infer Amplicon Sequence Variants (ASVs). Sequence quality profiles were inspected before filtering to optimize trimming parameters. Taxonomic assignment was performed using the Greengenes2 reference database (release 2022.10), and a phylogenetic tree was reconstructed using the q2-align-to-tree-mafft-fasttree pipeline. The ASV abundance table, taxonomic assignments, and phylogenetic tree were subsequently imported into R (version 4.3.2) for downstream analyses.

Potential confounding variables were evaluated using Microbiome Multivariable Association with Linear Models 2 (MaAsLin2), a statistical framework designed to identify associations between microbial features and clinical or phenotypic variables while accounting for potential confounding factors. Age, routine laboratory parameters, and psychological assessment scores (CDI-2 and MASC-2) were evaluated as additional potential confounding variables. Given the limited sample size, each variable was tested in a separate model. *p*-values were adjusted for multiple testing using the Benjamini–Hochberg false discovery rate (FDR) procedure.

Low-prevalence ASVs were filtered by retaining only taxa detected in at least 25% of samples. Data were normalized using Cumulative Sum Scaling (CSS) to account for the compositional nature of microbiome sequencing data.

Alpha diversity was evaluated using the Shannon, Simpson, and Chao1 indices. Statistical differences between groups were assessed using the Mann–Whitney U test. Beta diversity was calculated using Bray–Curtis and unweighted UniFrac distance matrices, visualized by Principal Coordinates Analysis (PCoA), and statistically evaluated by Permutational Multivariate Analysis of Variance (PERMANOVA).

Differentially abundant taxa were identified using Linear Discriminant Analysis Effect Size (LEfSe), applying a significance threshold of *p* < 0.05 and an LDA score > 3.

### 2.8. Predicted Functional Profiling of the Gut Microbiota

The predicted functional profile of the gut microbiota was inferred using PICRUSt2 [16], which predicts MetaCyc pathway abundances from the ASV table. Predicted pathway profiles were explored by principal component analysis (PCA), and the pathways with the highest absolute loading values were displayed to facilitate the biological interpretation of sample separation. Differential abundance of predicted MetaCyc pathways was assessed using MaAsLin2, with study group (AN vs. HC) as the main variable and age as a covariate. *p*-values were adjusted for multiple testing using the Benjamini–Hochberg false discovery rate (FDR), and pathways with an adjusted q-value < 0.05 were considered statistically significant.

### 2.9. Multivariate Analyses

To further characterize microbial community structure and identify bacterial signatures associated with severe anorexia nervosa, both unsupervised and supervised multivariate analyses were performed.

Principal component analysis (PCA) was carried out using the prcomp function implemented in R (vs. 4.5.1), whereas partial least squares discriminant analysis (PLS-DA) was performed using the mixOmics package.

To evaluate model robustness and exclude potential overfitting, PLS-DA models were validated by 200-permutation testing using the ropls package.

Only bacterial taxa with absolute loading coefficients between 0.10 and 0.30 and Variable Importance in Projection (VIP) scores ≥ 1 were retained for visualization and biological interpretation.

The predictive performance of the PLS-DA models was assessed using the Root Mean Square Error (RMSE), the coefficients of determination (R^2^ and Q^2^), and the Area Under the Receiver Operating Characteristic Curve (AUROC).

### 2.10. Statistical Analysis

Longitudinal changes in serum and urinary biomarkers, body mass index (BMI), and heart rate (HR) were assessed using linear mixed-effects models (LMMs), with time included as a fixed effect and subject as a random intercept. Statistical significance was evaluated using F-tests with Satterthwaite’s approximation, and *p*-values were adjusted for multiple testing using the Benjamini–Hochberg false discovery rate (FDR). Pairwise comparisons between time points were performed using estimated marginal means with Benjamini–Hochberg adjustment. Longitudinal trajectories were visualized using spaghetti plots.

### 2.11. Ethics Statement

The study was conducted according to the guidelines of the Declaration of Helsinki, and approved by the Institutional Review Board of Bambino Gesù Children’s Hospital, IRCCS (Protocol 3678_OPBG_2025; date of approval: 9 September 2025). Fecal samples from healthy controls were obtained from the Microbiome Special Collection of the OPBG BBMRI biobank, established through the EUBIOM Project and approved by the same Ethics Committee (Protocol No. 2839_OPBG_2022; Approval No. 1076, 26 July 2022; CE Version 1.0, 05/09/2022, EU-BIOM/2839_OPBG_2022).

Written informed consent was obtained from all participants and/or their parents or legal guardians before study enrolment.

## 3. Results

### 3.1. Baseline Clinical Characteristics

The baseline demographic, clinical and biochemical characteristics of the study cohort are summarized in Table 1. The study cohort comprised 26 adolescents with severe anorexia nervosa, with a marked female predominance (25/26, 96.2%; 1 male, 3.8%) and a median age of 15 years (IQR 14–16). At hospital admission (T0), participants presented the characteristic clinical phenotype of severe anorexia nervosa, with a median BMI of 14.1 kg/m^2^ (IQR 13.5–15.0), corresponding to a median BMI z-score of −3.27 (IQR −4.42 to −2.73) and to a median %mBMI of 70.9% (IQR 66.4–74.2) for age and sex (Table 1). Twenty-four out of the 26 participants (92.3%) had a BMI below the 5th percentile for age and sex, and malnutrition was graded as severe in 9 patients (34.6%), moderate in 15 (57.7%) and mild in 2 (7.7%). Resting blood pressure, heart rate, and body temperature were at or below the lower limit of the physiological range. At the baseline, 15.4% of patients were receiving psychotropic medications, including aripiprazole, sertraline, and fluoxetine, while neuropsychiatric comorbidities were documented in 19.2% of the cohort, mainly comprising depressive symptoms, obsessive-compulsive traits, and anxiety-depressive disorders.

Routine hematological and biochemical parameters were generally preserved despite severe malnutrition. White blood cell and neutrophil counts tended to be at the lower end of the physiological range, whereas hemoglobin, platelet count, renal function, electrolyte balance, and mineral homeostasis were largely maintained. Liver enzymes displayed substantial inter-individual variability, with occasional elevations in AST and ALT. Micronutrient status was heterogeneous across the cohort, particularly for vitamins C and D and ferritin concentrations (Table 1).

The HC group included 26 pediatric subjects, with a median age of 14 years (IQR: 12–15 years). Females accounted for 69.2% of the HC population. The median BMI was 19.4 kg/m^2^ (IQR: 18.4–20.0 kg/m^2^).

### 3.2. Longitudinal Clinical and Psychological Assessment

Twenty-one out of the 26 enrolled participants completed the three-month (T2) follow-up, whereas five participants were lost to follow-up because they were transferred to other healthcare facilities or did not attend the scheduled visit. Longitudinal analyses included all available observations through the use of linear mixed-effects models.

Longitudinal changes in clinical characteristics were evaluated using linear mixed-effects models with time as a fixed effect and participant as a random effect. Both BMI and heart rate changed significantly over the observation period (BMI: *p* < 0.001; heart rate: *p* < 0.001) (Figure 1, Table S1). Post hoc analyses (Table S2) showed a progressive increase in BMI across all study time points, with significant differences between T0 and T_1_ (*p* < 0.001), T0 and T2 (*p* < 0.001), and T1 and T2 (*p* = 0.003). Heart rate also increased significantly from the baseline to T1 (*p* < 0.001) and from the baseline to T2 (*p* < 0.001), whereas no significant difference was observed between the two follow-up visits (*p* = 0.173).

Longitudinal psychological assessment was performed using the CDI-2 and MASC-2 questionnaires at the three study time points (Table 2). At the baseline (T0), 34.8% of participants were classified within the clinically significant or severely clinical categories for depressive symptoms according to the CDI-2 total score, whereas 52.2% showed clinically significant or severely clinical anxiety symptoms based on the MASC-2 total score. At T1, the proportion of participants within the normal range increased for both the CDI-2 (73.9%) and MASC-2 (60.9%) total scores. At T2, most evaluated participants remained within the normal range for depressive symptoms (68.8%), whereas severely clinical anxiety scores were still observed in 40.0% of participants completing the assessment.

Both the CDI-2 and MASC-2 total scores showed significant longitudinal changes in linear mixed-effects models (CDI-2: *p* = 0.0018, FDR = 0.0037; MASC-2: *p* = 0.0075, FDR = 0.0075) (Figure 1, Tables S1 and S2). Post hoc analyses showed a significant reduction in CDI-2 scores from T0 to T1 (*p* = 0.021) and from T0 to T2 (*p* = 0.0025), whereas the T1–T2 comparison was not significant (*p* = 0.197). For MASC-2, a significant reduction was observed between T0 and T2 (*p* = 0.008), while the T0–T1 (*p* = 0.112) and T1–T2 (*p* = 0.115) comparisons were not significant. Overall, these results indicate significant temporal changes in depressive and anxiety symptom scores during the observation period, with the clearest differences observed between the baseline and the three-month follow-up. Because of the reduced number of available questionnaires at T2, these results should be interpreted descriptively.

Also, serum creatinine, phosphorus, magnesium, and ferritin showed significant longitudinal changes during nutritional rehabilitation. Serum creatinine decreased significantly over time, with a significant reduction observed between the baseline (T0) and the 3-month follow-up (T2). In contrast, serum phosphorus and magnesium increased significantly from the baseline to the first month of treatment (T1) and remained stable thereafter. Similarly, serum ferritin decreased significantly during the first month and remained unchanged between T1 and T2. No significant longitudinal changes were observed for the remaining routine hematological parameters (Tables S1 and S2).

### 3.3. Gut Microbiota Ecology in Patients with AN and Healthy Controls

High-throughput sequencing generated a total of 3067 amplicon sequence variants (ASVs) that passed quality control. After denoising, chimera removal, and taxonomic filtering, the mean sequence length was 409 bp (range: 233–429 bp). Sequencing depth averaged 28,614 reads per sample, ranging from 2552 to 182,007 reads, indicating adequate coverage for downstream microbial diversity and taxonomic analyses.

To investigate whether severe anorexia nervosa was associated with alterations in gut microbial diversity, alpha and beta diversity metrics were compared between patients at the baseline (T0) and healthy controls (Figure 2).

Alpha diversity analysis revealed no significant differences between groups according to the Shannon–Wiener (*p* = 0.54) and Simpson (*p* = 0.27) indices. In contrast, the Chao1 index, which estimates community richness, was significantly lower in AN patients than in healthy controls (*p* = 0.00013) (Figure 2A–C).

Beta diversity analyses demonstrated a clear separation between AN and HC microbial communities. Principal coordinate analysis (PCoA) based on both Bray–Curtis and unweighted UniFrac distance matrices showed distinct clustering of the two groups (Figure 2D,E). Pairwise PERMANOVA confirmed significant differences in overall microbial community structure using both Bray–Curtis distances (R^2^ = 0.0724, adjusted *p* = 0.001) and unweighted UniFrac distances (R^2^ = 0.0540, adjusted *p* = 0.001).

A descriptive overview of the gut microbiota composition at the phylum and family levels is provided in Supplementary Figure S1. Overall, the fecal microbiota of AN patients was dominated by the same major bacterial phyla and families observed in the healthy controls, although differences in their relative abundances were evident.

To further characterize taxonomic differences between groups, supervised and unsupervised multivariate analyses were performed at the genus level (Figure 3). Partial Least Squares Discriminant Analysis (PLS-DA) showed a clear separation between adolescents with AN and healthy controls (Figure 3A). The loading plot and Variable Importance in Projection (VIP) scores identified the bacterial genera that contributed most to group discrimination, with all reported genera displaying VIP values > 1 (Figure 3B,C). The robustness of the PLS-DA model was supported by cross-validation (AUROC = 0.98, RMSE = 0.184, R^2^ = 0.872, Q^2^ = 0.699; *p* ≤ 0.05).

Consistent with these findings, unsupervised the PCA also revealed a partial separation between AN and HC samples, with the first two principal components explaining 21.0% and 10.78% of the total variance, respectively (Figure 3D).

Differential abundance analysis using LEfSe identified 19 bacterial genera that significantly discriminated the two groups (Figure 3E,F). Compared with healthy controls, adolescents with AN were enriched in *Blautia*_A_141781, *Romboutsia*_B, *Clostridium*_T, *Anaerostipes*, *Mediterraneibacter*_A_155507, *Vescimonas*, f__*Acutalibacteraceae*, *Anaerobutyricum*, *Turicibacter*, and *Copromorpha*, whereas healthy controls showed higher relative abundances of ER4, *Prevotella*, *Ruminococcus*_E, *Fimetecus*, *Agathobacter*_164117, *Gemmiger*_A_73129, *Bacteroides*_H, *Phocaeicola*_A_858004, and *Akkermansia*.

To assess potential confounding effects on gut microbiota composition, multivariable association analyses were performed using MaAsLin2, considering age, routine laboratory parameters, and psychological assessment scores (CDI-2 and MASC-2) as potential confounding variables. After FDR correction, Ruminococcus_E was the only bacterial taxon showing a significant association with both disease status and age, whereas the remaining differentially abundant taxa were associated exclusively with disease status (Figure S2). Among the additional potential confounding variables examined, significant associations (q < 0.05) were identified between *Intestinibacter* and lymphocyte counts, *Romboutsia*_B and lymphocyte counts, *Escherichia*_710834 and blood urea nitrogen levels, *Akkermansia* and vitamin C levels, and *Alistipes*_A_871400 and blood glucose concentrations (Table S3). To complement the taxonomic findings, the predicted functional profile of the gut microbiota was assessed using PICRUSt2 (Supplementary Figure S4). Predicted functional profiling revealed a partial separation between the AN and HC samples (Supplementary Figure S4A). Differential abundance analysis performed with MaAsLin2, adjusting for age, identified 19 predicted MetaCyc pathways significantly associated with the study group (Supplementary Figure S4B). Adolescents with AN showed altered representation of predicted pathways involved in amino acid metabolism (including arginine, ornithine, and lysine metabolism), purine and pyrimidine metabolism, carbohydrate degradation, microbial fermentation, peptidoglycan biosynthesis, and cofactor biosynthesis, whereas HC showed a higher predicted abundance of pathways related to pyruvate fermentation to propionate, central energy metabolism (tricarboxylic acid cycle), and lipid biosynthesis.

In summary, at hospital admission, the gut microbiota of adolescents with severe AN differed from that of the healthy controls in bacterial richness, overall taxonomic composition, and predicted metabolic potential, whereas diversity and evenness were comparable between groups.

### 3.4. Longitudinal Changes in Gut Microbiota Diversity

Gut microbial diversity was compared across the three study time points (T0, T1, and T2) to evaluate temporal changes in microbial community structure (Figure 4). No significant differences in alpha diversity were observed across the three study time points according to the Shannon–Wiener, Simpson, or Chao1 indices (all *p* > 0.05) (Figure 4A–C).

In contrast, beta diversity analysis based on Bray–Curtis distances revealed modest but significant longitudinal changes in microbial community composition (Figure 4D). Pairwise PERMANOVA identified significant differences between all pairs of time points (all adjusted *p* < 0.05), whereas no significant differences were detected using unweighted UniFrac distances (Figure 4D,E).

The overall taxonomic composition of the gut microbiota remained largely stable throughout the study period, with the same dominant bacterial phyla and families detected at T0, T1, and T2, despite gradual changes in the relative abundance of individual taxa (Supplementary Figure S3).

To identify bacterial taxa associated with temporal changes during follow-up, differential abundance analysis was performed using LEfSe (Figure 5). Eight bacterial genera were identified as significantly different across the three study time points. *Escherichia*_710834, *Turicibacter*, and *Eggerthella* were enriched at the baseline (T0), whereas *Faecalibacterium*, *Gemmiger*_A_73129, *Agathobacter*_164117, *Acetatifactor*, and members of the *Lachnospiraceae* family were enriched at T2 (Figure 5A). The longitudinal abundance profiles of these taxa are shown in Figure 5B and indicate progressive shifts in the relative abundance of selected bacterial genera over the course of follow-up.

In summary, over three months of nutritional rehabilitation, the gut microbiota showed changes in the relative abundance of individual taxa, without measurable changes in alpha diversity or in the phylogenetic structure of the community.

### 3.5. Longitudinal Changes in Host Biomarkers

To characterize the host biological profile associated with severe anorexia nervosa, serum and urinary biomarkers related to microbial metabolism, intestinal barrier integrity, mucosal immunity, inflammatory response, gut–brain, and gastrointestinal signaling were assessed at the baseline (Table 3). Baseline concentrations of all serum and urinary biomarkers are reported in Table 3, together with their longitudinal changes.

Longitudinal changes in host biomarkers were assessed using linear mixed-effects models including time as a fixed effect and participant as a random effect (Figure 6, Table S4). Significant temporal changes were observed for urinary nitric oxide (*p* = 8.2 × 10^−5^), urinary indican (*p* = 3.6 × 10^−4^), urinary serotonin (*p* = 0.004), and serum occludin (*p* = 0.005), whereas bile acids, short-chain fatty acids, serum IgA, lipopolysaccharide, and haptoglobin did not show significant longitudinal variation. After FDR correction, the temporal effects remained significant for nitric oxide, indican, serotonin, and occludin (FDR < 0.05).

Post hoc analyses (Table S5) showed that urinary indican increased progressively over the observation period, with a significant difference between T0 and T2. Urinary serotonin increased significantly at T2 compared with T0, whereas nitric oxide levels were significantly higher at T1 and T2 than at the baseline, with no significant difference between the two follow-up visits. Serum occludin showed a transient decrease at T1 followed by a significant increase at T2 compared with T1, while no significant difference was observed between T0 and T2.

In summary, biomarkers related to microbial protein fermentation, gut–brain signaling and epithelial barrier turnover changed significantly during follow-up, whereas markers of mucosal immunity, systemic inflammation, and bile acid and short-chain fatty acid metabolism remained stable.

## 4. Discussion

This is one of the few prospective pediatric studies integrating gut microbiota profiling, host-derived biomarkers, routine clinical parameters and psychological assessment during nutritional rehabilitation, providing a comprehensive picture of host–microbiota interactions in severe anorexia nervosa.

In this prospective longitudinal study of adolescents with severe AN, gut microbiota dysbiosis at hospital admission was followed by only partial microbial remodeling during three months of nutritional rehabilitation, alongside measurable changes in selected host-derived biomarkers. By integrating microbiota profiling with routine clinical, biochemical and psychological data, our findings extend previous cross-sectional observations and provide longitudinal insight into how the gut microbiota and host physiology evolve together during renutrition.

At the baseline, adolescents with severe AN showed a distinct gut microbial ecology compared with the healthy controls. Although overall community diversity and evenness were preserved, bacterial richness was reduced, and both taxonomic composition and phylogenetic structure differed from those of the healthy controls. These findings are consistent with previous reports describing altered gut microbial ecosystems in patients with AN, characterized by the depletion of beneficial SCFA-producing taxa and the enrichment of bacteria adapted to nutrient deprivation [8,9,10]. Rather than examining each differentially abundant genus individually, these shifts are more informative when interpreted by functional category: several taxa enriched in AN, such as *Clostridium*_T, *Anaerobutyricum* and *Turicibacter*, are broadly associated with mucin degradation and adaptation to nutrient-poor, low-energy environments, whereas genera enriched in HC, including *Prevotella*, *Akkermansia* and *Agathobacter*, are recognized SCFA producers and markers of a fiber-fermenting, eubiotic community [8,17].

Predicted functional profiling of the gut microbiota (PICRUSt2, Supplementary Figure S4) provided additional information on the predicted functional profile associated with the observed taxonomic differences. AN-enriched predicted pathways involved in amino acid metabolism, purine and pyrimidine metabolism, peptidoglycan maturation, and cofactor biosynthesis may indicate differences in the predicted metabolic potential of the intestinal microbiota between groups. These findings are consistent with previous reports describing alterations in microbial metabolic profiles in AN [17,18]. Conversely, the higher predicted abundance in HC of pathways related to pyruvate fermentation to propionate and to central energy and lipid metabolism further suggests differences in the predicted functional profile between groups. These findings are broadly consistent with previous reports of altered SCFA production in AN [18,19], although circulating SCFA concentrations in our cohort did not show significant longitudinal changes. As these functional profiles are inferred from 16S rRNA gene data rather than measured directly, they should be interpreted as hypothesis-generating estimates of microbial metabolic potential rather than as evidence of confirmed changes in microbial functional activity.

During the three months of nutritional rehabilitation, the gut microbiota showed only partial remodeling. Changes were confined to the relative abundance of taxa already present in the community, without measurable changes in alpha diversity or in its phylogenetic structure. The progressive enrichment of *Faecalibacterium*, *Gemmiger*_A, and *Agathobacter*, together with other Lachnospiraceae members, at T2, alongside the depletion of *Escherichia*, *Turicibacter* and *Eggerthella* observed at the baseline, points toward a partial recovery of SCFA-producing taxa during refeeding. These findings are in line with previous longitudinal studies reporting that gut dysbiosis in AN persists, at least partially, despite short-term weight restoration, and that full microbial recovery may require longer observation periods or additional targeted interventions [4,11]. The absence of any increase in alpha diversity over three months contrasts with reports of the partial recovery of richness after weight restoration in adult inpatients [11]; this discrepancy may reflect the shorter follow-up of our study, the younger age of our cohort, and differences in the caloric density and composition of the refeeding protocols.

Beyond microbiota composition, several host-derived biomarkers of intestinal barrier function, microbial metabolism and gut–brain signaling showed significant longitudinal changes during nutritional rehabilitation, suggesting a dynamic host response accompanying clinical recovery. Urinary indican, a marker of intestinal bacterial protein fermentation, and the gut–brain signaling markers serotonin and nitric oxide increased during follow-up, whereas serum occludin followed a biphasic trajectory. The temporal changes in these biomarkers occurred alongside the microbial shifts observed during nutritional rehabilitation, including the progressive enrichment of *Faecalibacterium*, *Gemmiger*_A, *Agathobacter*, and other *Lachnospiraceae* members at T2, together with the depletion of *Escherichia*, *Turicibacter* and *Eggerthella*. Given the recognized involvement of the gut microbiota and its metabolites in intestinal fermentation, enterochromaffin and nitrergic signaling, and epithelial barrier homeostasis, the concurrent changes in microbial composition, microbial metabolic markers, and occludin may reflect interconnected aspects of the host response to nutritional rehabilitation. However, the present data do not allow a direct causal relationship between specific microbial taxa and these host-derived biomarkers to be established [5,6]. In contrast, bile acids, SCFAs, IgA, LPS, and haptoglobin remained relatively stable across the observation period, suggesting that microbial and host-related pathways may recover at different rates during nutritional rehabilitation or that longer follow-up may be required to detect broader metabolic and immune changes.

Comparison with values reported in healthy populations suggests that several of these biomarkers were substantially altered at the baseline: urinary indican concentrations, reportedly around 10–25 mg/L in healthy individuals [20], were higher in our cohort, consistent with altered intestinal protein fermentation and dysbiosis; serum occludin concentrations, reportedly around 0.13 ± 0.02 ng/mL in healthy individuals [21], were markedly higher than the published reference values, potentially reflecting increased epithelial turnover or barrier dysfunction, although differences in ELISA kits and assay calibration across studies limit the direct comparability of absolute values; and circulating SCFA concentrations, reportedly around 60.0 ± 45.9 µmol/L in healthy individuals [22], were lower than the reported reference values, in keeping with the reduced predicted fermentative capacity of the AN gut microbiota discussed above. These comparisons should be regarded as descriptive rather than confirmatory, given that published studies differ in analytical platforms, biological matrices, normalization procedures, and reporting units.

Taken together, these findings suggest that host-derived biomarkers of intestinal barrier function and microbial metabolism may represent complementary, minimally invasive tools for monitoring biological recovery during nutritional rehabilitation in adolescents with AN. In practical terms, urinary indican, serotonin, and nitric oxide can be obtained from a first morning void, and serum occludin from the blood sample already drawn at routine monitoring visits, so that they can be measured repeatedly without additional burden for the patient and without the cost and turnaround time of sequencing. Because they changed measurably over three months, whereas alpha diversity did not, they may capture aspects of intestinal recovery that anthropometric indices alone do not, and could be used alongside BMI to identify patients in whom barrier function and microbial metabolic activity lag behind weight restoration [19]. Any such application, however, would first require validation in larger independent cohorts, a creatinine-normalized measurement of urinary analytes, and the definition of age- and sex-specific pediatric reference intervals. Integrated profiling of the gut microbiota and host-derived biomarkers may improve our understanding of the biological mechanisms underlying AN and help identify novel, microbiota-targeted therapeutic strategies. Future interventional studies, ideally multicenter and of longer duration, will be needed to determine whether modulation of the gut microbiota can improve clinical outcomes in this population.

## 5. Limitations

The study included a relatively small number of participants from a single tertiary pediatric center, which may limit the generalizability of the findings.

The follow-up period was limited to three months; longer longitudinal studies are needed to determine whether microbiota and host-derived biomarkers fully recover after nutritional rehabilitation.

Healthy controls were available only for microbiota comparisons, whereas longitudinal host biomarker measurements were performed exclusively in patients with anorexia nervosa.

Nutritional rehabilitation combined oral intake, oral nutritional supplements, and when clinically indicated, nasogastric enteral feeding. Since the route and composition of nutrient delivery may themselves influence gut microbial composition and fermentative activity, and since the feeding route delivered to each patient was not systematically recorded, the contribution of feeding modality to the observed microbiota changes could not be assessed.

Finally, enrolment was based on an absolute BMI threshold of 16 kg/m^2^, which derives from adult criteria; although all participants were severely malnourished when assessed with age- and sex-specific indices, and although severity was corroborated by the clinical criteria for inpatient admission, BMI-based severity specifiers have limited clinical validity in this age range [15], and a prospective definition anchored to BMI percentiles or to %mBMI would be preferable in future studies.

Urinary biomarkers were reported as absolute concentrations because urinary creatinine measurements were not available for normalization. Therefore, the potential influence of urine dilution on biomarker concentrations cannot be completely excluded.

Direct comparison with published healthy reference values should be interpreted cautiously because many studies report measurements obtained using different analytical platforms, assay kits, biological matrices, normalization strategies (e.g., creatinine-normalized concentrations), and measurement units.

Standardized reference intervals for several host-derived biomarkers are currently unavailable, particularly in pediatric populations, making interpretation across studies challenging.

The observational design does not allow for causal relationships between gut microbiota alterations, host-derived biomarkers, and clinical outcomes to be established.

Future multicenter studies including larger cohorts, longer follow-up, standardized biomarker methodologies and functional multi-omics approaches will be necessary to validate and extend these findings.

## 6. Conclusions

In conclusion, this prospective longitudinal study shows that adolescents with severe AN present significant gut microbiota dysbiosis at hospital admission, characterized by reduced bacterial richness and altered taxonomic and predicted functional profiles, with only partial microbial remodeling during three months of nutritional rehabilitation. These changes were accompanied by significant longitudinal variations in host-derived biomarkers of intestinal barrier function and microbial metabolism, and by a limited number of associations between the gut microbiota, host-derived biomarkers, and routine clinical parameters. Taken together, these findings support the gut microbiota and its functional and metabolic correlates as relevant, integrated components of the pathophysiology of severe AN in adolescence, and suggest that host-derived biomarkers may serve as complementary, minimally invasive tools for monitoring biological recovery during nutritional rehabilitation. Larger, multicenter and longer-term studies, ideally with interventional designs, are needed to confirm these findings and to explore whether microbiota-targeted strategies can improve clinical outcomes in this population.

## Figures and Tables

**Figure 1:**
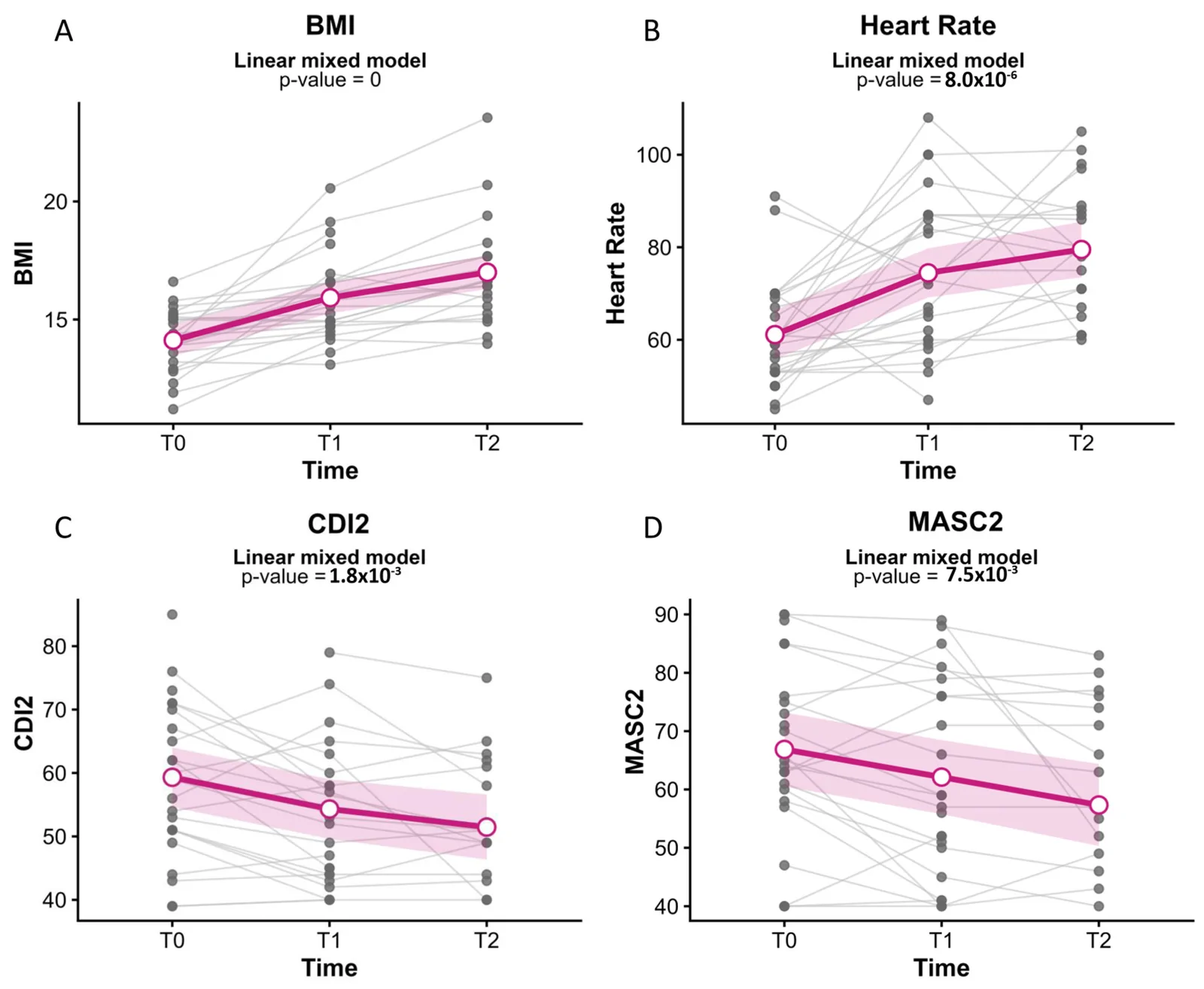
Longitudinal changes in body mass index (BMI), heart rate CDI-2, and MASC-2 in adolescents with severe anorexia nervosa. Individual trajectories are shown as gray lines connecting repeated measurements obtained at the baseline (T0), after one month (T1), and after three months (T2). The pink lines represent the estimated mean trajectories, pink circles represent the estimated marginal means derived from the linear mixed-effects models, and the shaded areas indicate the corresponding 95% confidence intervals. Temporal changes were assessed using linear mixed-effects models with time as a fixed effect and participant as a random effect. Overall *p*-values are reported in each panel.

**Figure 2:**
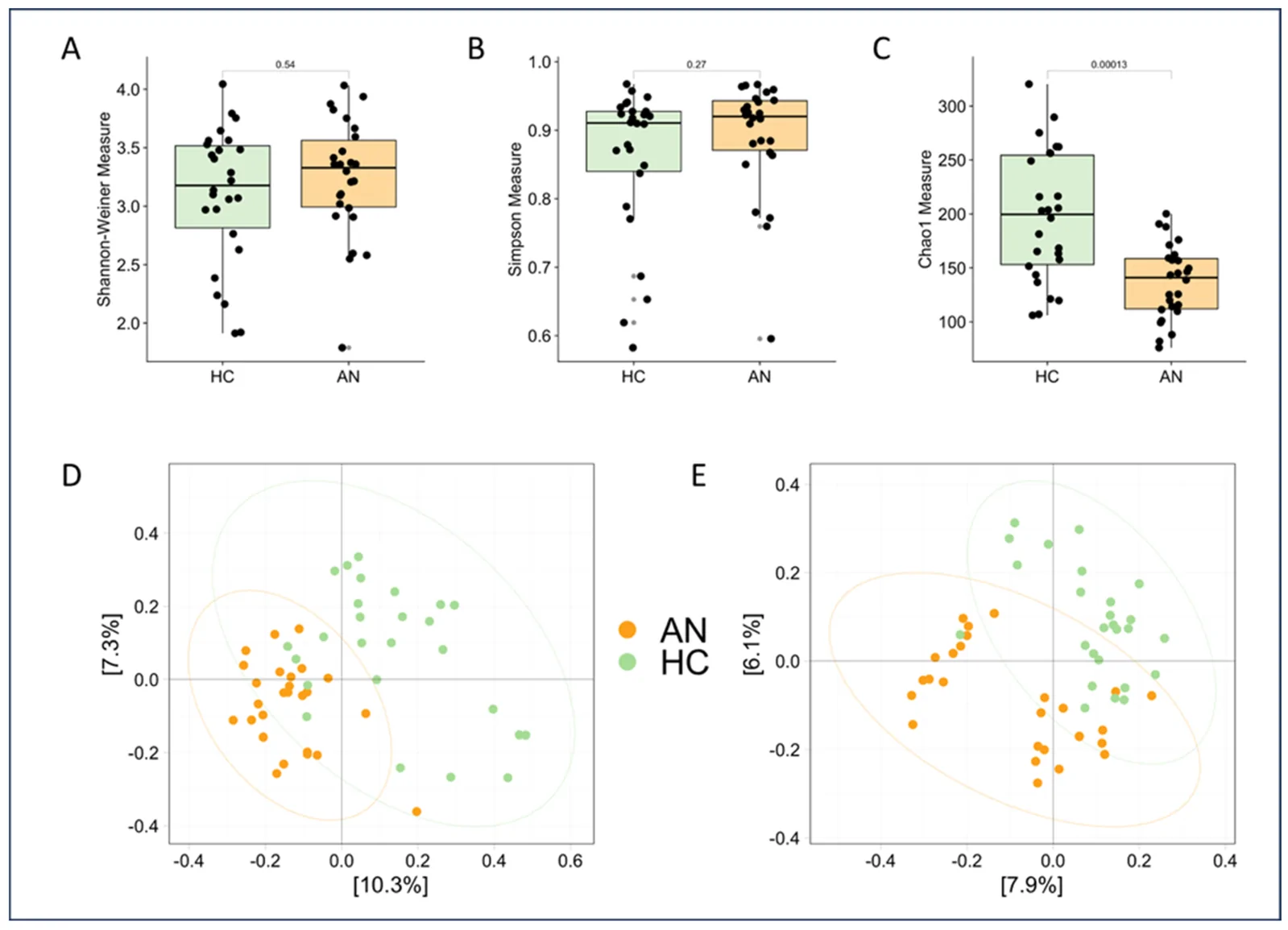
Gut microbiota alpha and beta diversity in adolescents with severe anorexia nervosa (AN) at the baseline (T0) and healthy controls (HC). Alpha diversity was evaluated using the Shannon–Wiener (**A**), Simpson (**B**), and Chao1 (**C**) indices. Boxplots display the median and interquartile range, with individual samples overlaid as dots. Group differences were assessed using the Mann–Whitney U test. No significant differences were observed for the Shannon–Wiener or Simpson indices, whereas the Chao1 index was significantly lower in AN patients than in HC, indicating reduced microbial richness (*p* = 0.00013). Beta diversity was assessed using Bray–Curtis (**D**) and unweighted UniFrac (**E**) distance matrices and visualized by principal coordinate analysis (PCoA). Pairwise PERMANOVA revealed significant differences in overall microbial community composition between AN and HC using both Bray–Curtis (R^2^ = 0.0724, adjusted *p* = 0.001) and unweighted UniFrac (R^2^ = 0.0540, adjusted *p* = 0.001) distance metrics.

**Figure 3:**
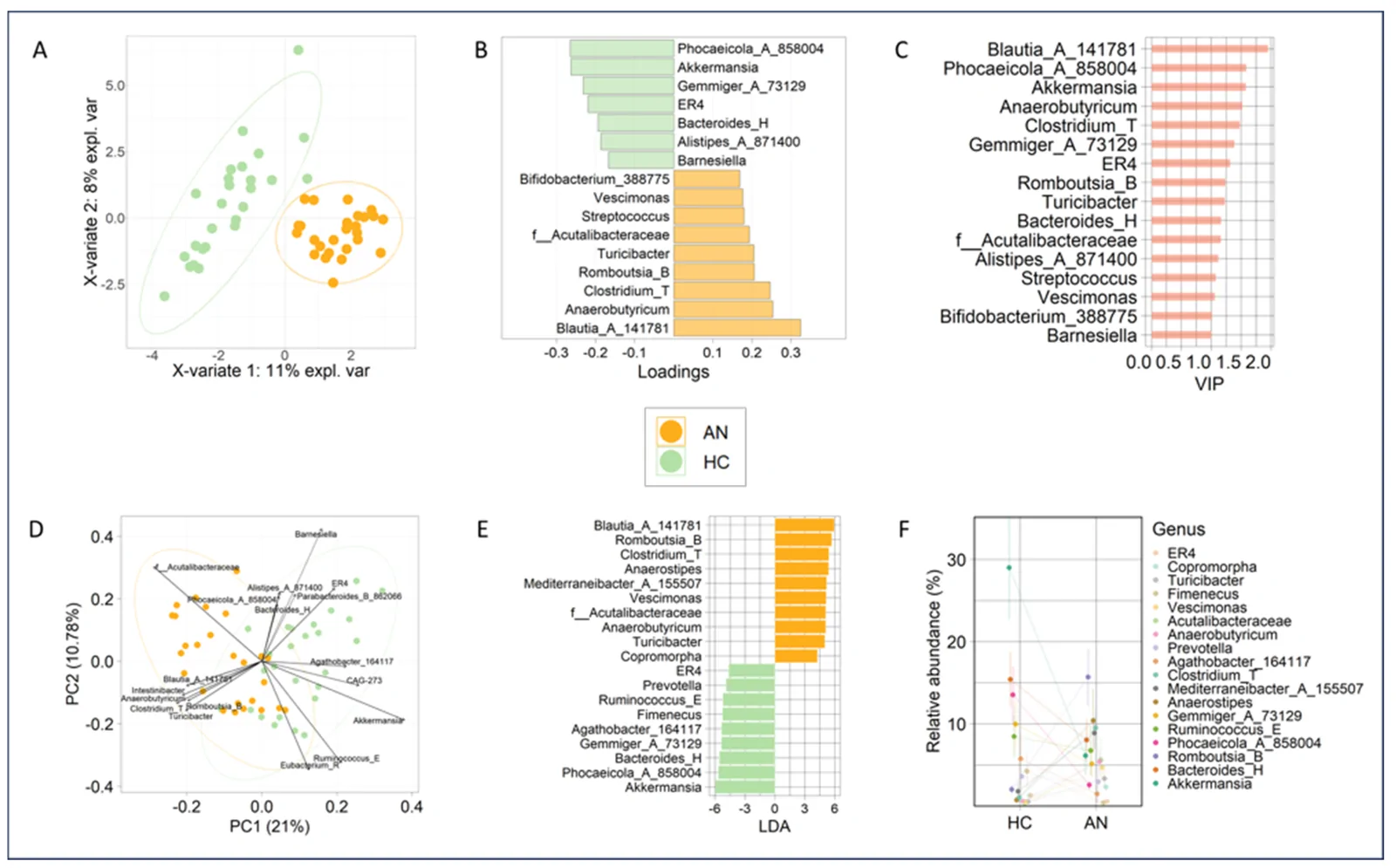
Differential abundance and multivariate analyses of the gut microbiota at the genus level in adolescents with severe anorexia nervosa (AN) at the baseline (T0) and healthy controls (HC). Supervised partial least squares discriminant analysis (PLS-DA) revealed a clear separation between AN and HC (**A**). The corresponding loading plot (**B**) and Variable Importance in Projection (VIP) scores (**C**) identified the bacterial genera contributing most to group discrimination (VIP > 1). Model performance was evaluated by cross-validation (AUROC = 0.98, RMSE = 0.184, R^2^ = 0.872, Q^2^ = 0.699; *p* ≤ 0.05). Unsupervised principal component analysis (PCA) showed a partial separation between AN and HC, with the first two principal components explaining 21.0% and 10.78% of the total variance, respectively (**D**). Linear discriminant analysis effect size (LEfSe) identified 19 differentially abundant bacterial genera, including ten enriched in AN (orange) and nine enriched in HC (green), with an LDA score > 3.0 (**E**). Panel (**F**) shows the relative abundances of the bacterial genera identified as significantly different by LEfSe.

**Figure 4:**
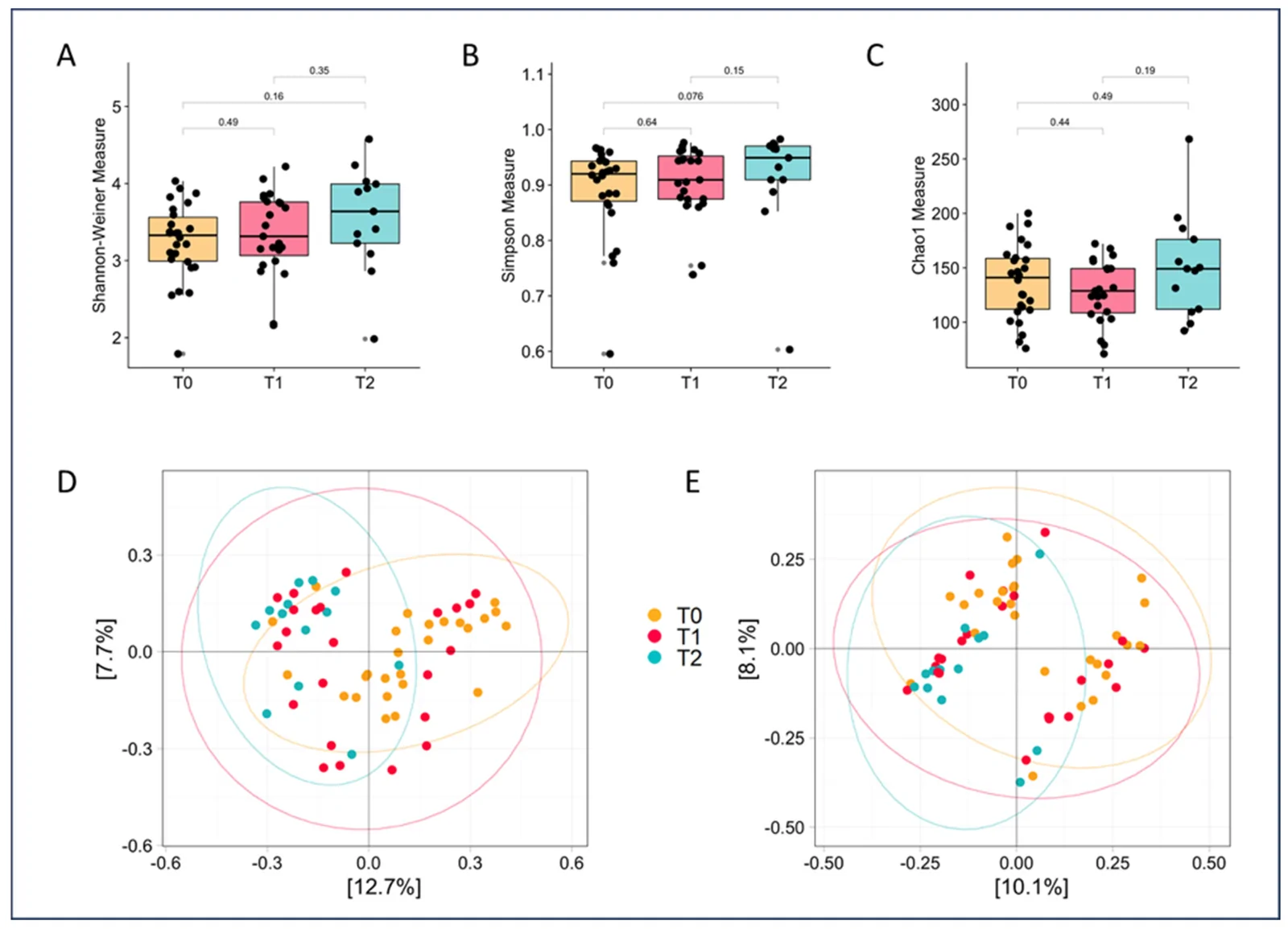
Longitudinal analysis of gut microbiota diversity in adolescents with severe anorexia nervosa across the three study time points. Alpha diversity was assessed at hospital admission (T0), after one month (T1), and after three months (T2) using the Shannon–Wiener (**A**), Simpson (**B**), and Chao1 (**C**) indices. No significant differences were observed among the three time points. Beta diversity was evaluated using Bray–Curtis (**D**) and unweighted UniFrac (**E**) distance matrices and visualized by principal coordinate analysis (PCoA). Pairwise PERMANOVA revealed significant differences in microbial community composition based on Bray–Curtis distances between T0 and T1 (R^2^ = 0.0337, adjusted *p* = 0.03), T0 and T2 (R^2^ = 0.0394, adjusted *p* = 0.04), and T1 and T2 (R^2^ = 0.0471, adjusted *p* = 0.03), whereas no significant differences were detected using unweighted UniFrac distances. Boxplots show the median and interquartile range, with individual samples overlaid as dots.

**Figure 5:**
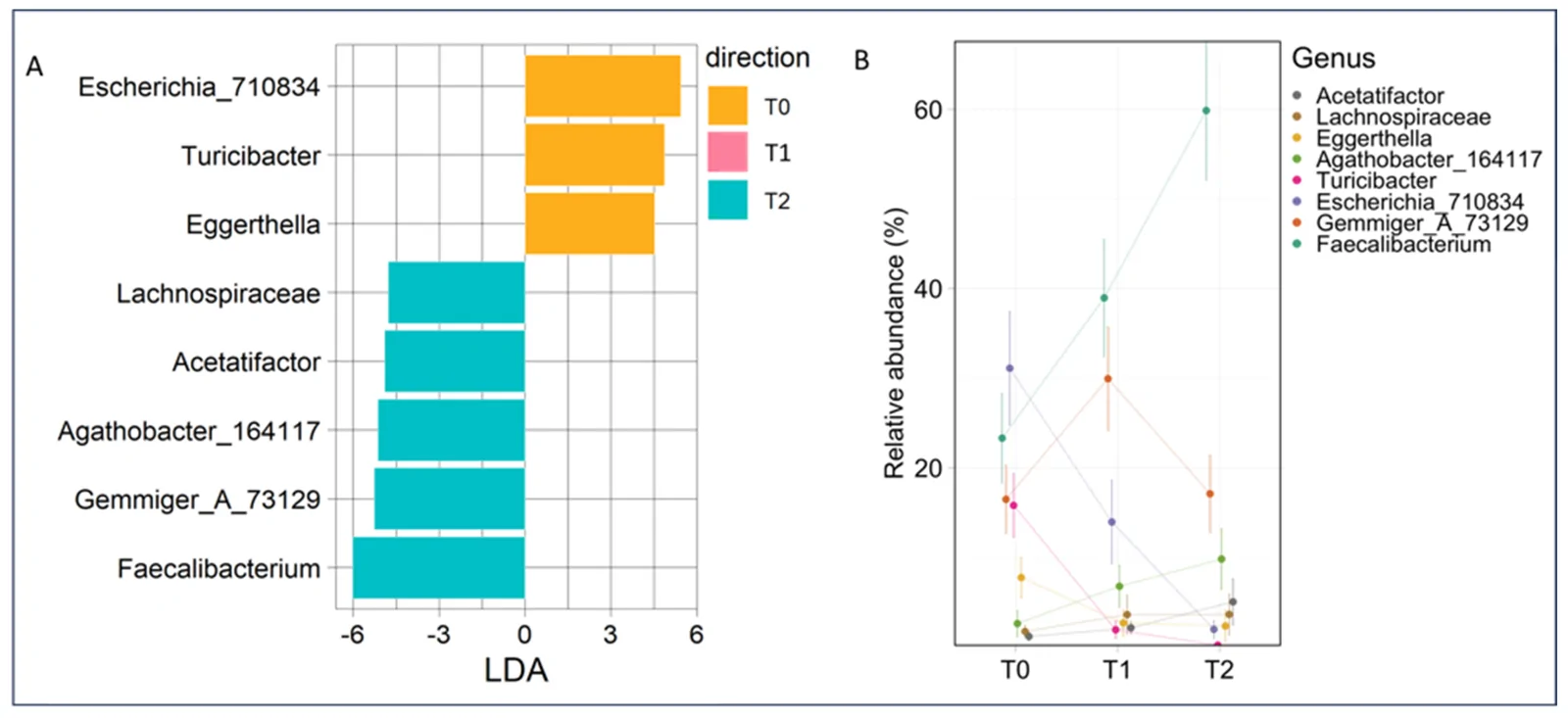
Longitudinal differential abundance analysis of the gut microbiota across the three study time points. Linear discriminant analysis effect size (LEfSe) identified eight bacterial taxa that differed significantly across T0, T1, and T2. T0-enriched taxa are shown in orange and T2-enriched taxa in teal; no taxa were statistically significant at T1 (pink) (**A**). Panel (**B**) shows the relative abundances of the taxa identified as significantly different by LEfSe across the three study time points.

**Figure 6:**
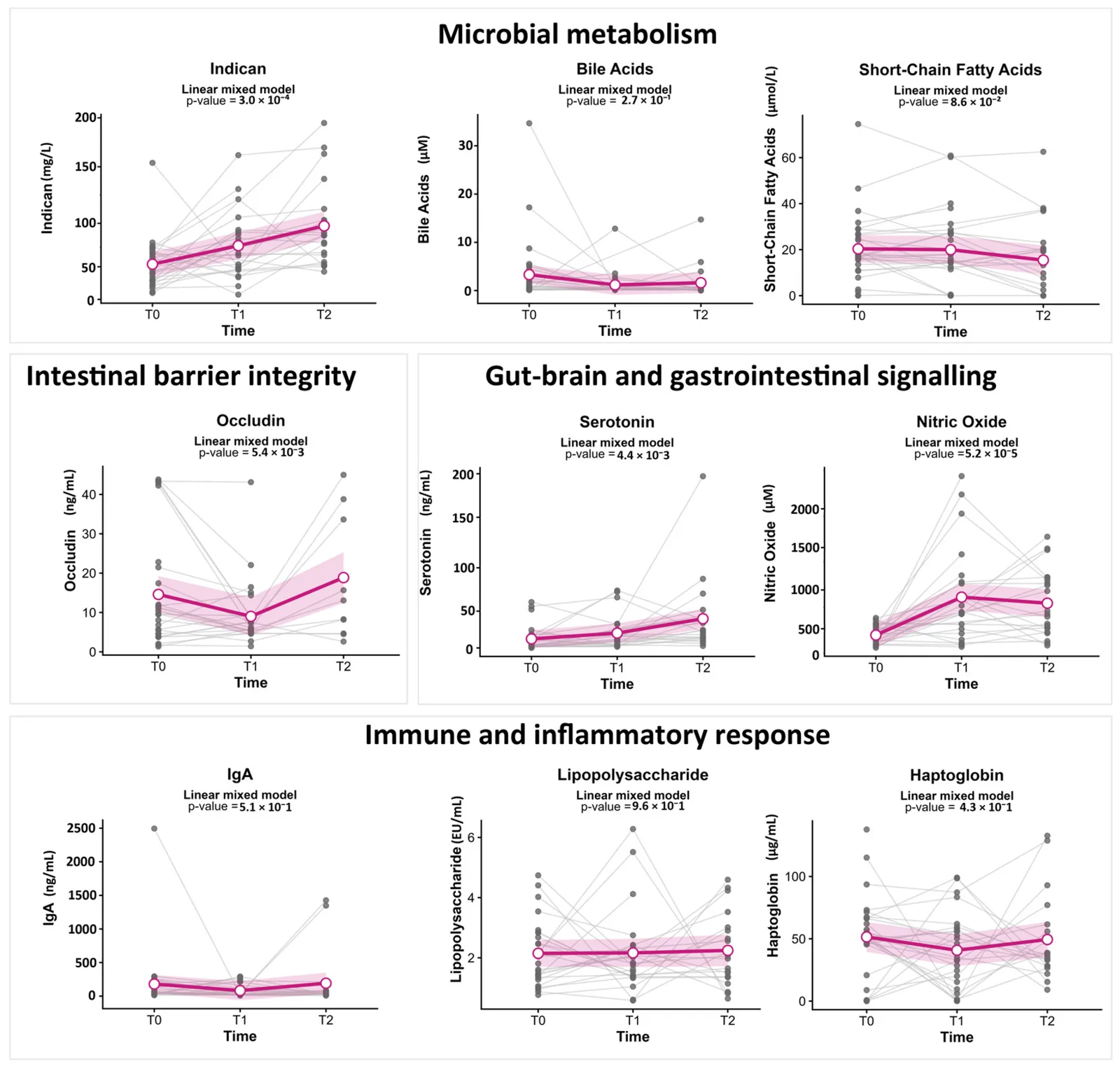
Longitudinal changes in host biomarkers during the observation period. Individual trajectories are shown as gray lines connecting repeated measurements obtained at the baseline (T0), after one month (T1), and after three months (T2). The pink lines represent the estimated mean trajectories, pink circles represent the estimated marginal means derived from the linear mixed-effects models, and the shaded areas indicate the corresponding 95% confidence intervals. The overall significance of temporal changes was assessed using linear mixed-effects models with time as a fixed effect and participant as a random effect; *p*-values are reported in each panel. Biomarkers showing significant longitudinal variation included urinary indican, urinary serotonin, urinary nitric oxide, and serum occludin, whereas bile acids, short-chain fatty acids, serum IgA, lipopolysaccharide, and haptoglobin did not change significantly over time.

**Table 1:** Baseline demographic and clinical characteristics of the study cohort (T0).

Patient Characteristics		
Age (years) Median (IQR)	15 (14–16)	
Female, n (%)	25 (96.2)	
Male, n (%)	1 (3.8)	
Disease duration (months)	8 (6.3–12.0)	
Previous nutritional treatment, n (%)	3 (11.5)	
Neuropsychiatric comorbidities, n (%)	5 (19.2)	
Concomitant psychopharmacological treatment, n (%)	4 (15.4)	
Anthropometric and clinical variables	Median (IQR)	
BMI (kg/m^2^)	14.1 (13.5–15.0)	
BMI z-score (CDC)	−3.27 (−4.42 to −2.73)	
Percentage of median BMI (%)	70.9 (66.4–74.2)	
Systolic blood pressure (mmHg)	103.5 (99.8–110.0)	
Diastolic blood pressure (mmHg)	70.0 (59.8–73.0)	
Heart rate (bpm)	59.0 (53.0–67.8)	
Body temperature (°C)	36.05 (36.0–36.2)	
Routine laboratory parameters	Reference range	Median (IQR)
White blood cells (×10^3^/mm^3^)	4.00–11.00	4.33 (3.72–5.42)
Neutrophils (×10^3^/mm^3^)	2.00–8.00	1.95 (1.63–2.97)
Lymphocytes (×10^3^/mm^3^)	0.80–4.73	1.77 (1.42–2.11)
Hemoglobin (g/dL)	12.0–16.0	13.5 (12.9–14.4)
Platelets (×10^3^/mm^3^)	150–450	192.5 (151.5–220.5)
Creatinine (mg/dL)	0.51–0.95	0.73 (0.64–0.88)
Blood urea nitrogen (mg/dL)	5–18	15.0 (11.8–18.0)
AST (U/L)	≤32	25.0 (19.0–31.8)
ALT (U/L)	≤33	19.0 (15.0–34.0)
Glucose (mg/dL)	60–100	75.5 (65.8–83.5)
Sodium (mmol/L)	136–145	139.5 (137.0–141.3)
Phosphorus (mg/dL)	2.5–4.8	3.5 (3.0–3.8)
Magnesium (mg/dL)	1.70–2.20	2.22 (2.12–2.34)
Total calcium (mg/dL)	8.4–10.2	9.5 (9.3–9.8)
Vitamin C (mg/L)	5–15	9.58 (3.81–17.63)
Vitamin D (ng/mL)	≥30 ‡	22.85 (17.03–27.95)
Ferritin (ng/mL)	30–400	259.2 (155.3–337.0)
Folate (ng/mL)	5.00–27.20	6.05 (3.92–6.45)

‡ Vitamin D: sufficiency was defined according to the Endocrine Society guidelines (30–100 ng/mL); insufficiency, 20–29 ng/mL; deficiency, <20 ng/mL. IQR: interquartile range; BMI: body mass index; AST: aspartate aminotransferase; ALT: alanine aminotransferase.

**Table 2:** Distribution of participants across clinical categories according to CDI-2 and MASC-2 total T-scores at the baseline (T0), one month (T1), and three months (T2).

Variable	Normal Value (%)	Mild Elevation (%)	Clinically Significant (%)	Severely Clinical (%)
CDI-2 Total Score (T_0_)	12 (52.2)	3 (13.0)	2 (8.7)	6 (26.1)
CDI-2 Total Score (T1)	17 (73.9)	2 (8.7)	2 (8.7)	2 (8.7)
CDI-2 Total Score (T2)	11 (68.8)	3 (18.8)	1 (6.3)	1 (6.3)
MASC-2 Total Score (T0)	6 (26.1)	5 (21.7)	2 (8.7)	10 (43.5)
MASC-2 Total Score (T1)	14 (60.9)	0 (0.0)	1 (4.3)	8 (34.8)
MASC-2 Total Score (T2)	7 (46.7)	1 (6.7)	1 (6.7)	6 (40.0)

Data are presented as value (%). Percentages were calculated using the number of participants with available psychometric assessment at each study time point. Participants with missing or incomplete questionnaires were excluded from the corresponding analysis. Clinical categories were defined as follows: normal (T-score 40–59), mild elevation (60–64), clinically significant (65–69), and severely clinical (≥70). CDI-2: Children’s Depression Inventory–2; MASC-2: Multidimensional Anxiety Scale for Children–2.

**Table 3:** Longitudinal changes in serum and urinary host biomarkers related to microbial metabolism, intestinal barrier integrity, mucosal immunity, and gut–brain and gastrointestinal signaling across the three study time points (T0, T1, T2).

Biomarker	T0, Median (IQR)	T1, Median (IQR)	T2, Median (IQR)
Indican (mg/L)	45.09 (26.78–69.80)	65.22 (39.15–71.71)	75.00 (51.82–92.59)
Bile acids (µM)	7.65 (1.00–22.53)	1.29 (1.00–4.20)	2.75 (1.00–6.93)
Serotonin (ng/mL)	47.41 (24.58–163.46)	101.92 (40.29–211.77)	240.07 (142.55–403.52)
Nitric oxide (µM)	507.52 (335.91–727.85)	1148.25 (596.57–1728.68)	1190.19 (554.88–1720.00)
Occludin (ng/mL)	9.53 (4.61–16.07)	6.20 (5.21–9.17)	17.60 (11.86–17.60)
SCFAs (µmol/L)	17.46 (15.73–26.75)	17.91 (14.29–27.53)	18.30 (6.98–21.21)
IgA (ng/mL)	378.60 (302.60–1324.62)	301.65 (217.77–1202.36)	323.78 (282.74–757.80)
LPS (EU/mL)	2.04 (1.35–2.68)	1.83 (1.37–2.31)	2.02 (1.37–2.93)
**Haptoglobin (µg/mL)**	50.45 (45.56–65.20)	35.15 (15.37–58.15)	37.67 (28.26–57.35)

Values are expressed as median (IQR). IQR: interquartile range; SCFAs: short-chain fatty acids; IgA: immunoglobulin A; LPS: lipopolysaccharide.

## Data Availability

The data presented in this study are available on request from the corresponding author. The data are not publicly available due to privacy and ethical restrictions, as they contain sensitive clinical and psychological information related to minor patients.

## Supplementary Materials

The following supporting information can be downloaded at: https://www.mdpi.com/article/10.3390/nu18193136/s1, Figure S1: Relative abundance of dominant bacterial taxa in adolescents with severe anorexia nervosa (AN) at the baseline (T0) and healthy controls (HC); Figure S2: Multivariable association analysis of differentially abundant bacterial taxa using MaAsLin2; Figure S3: Relative abundance of dominant bacterial taxa across the three study time points in adolescents with severe anorexia nervosa; Figure S4: Predicted functional profiling of the gut microbiota in adolescents with severe anorexia nervosa (AN) at the baseline (T0) and healthy controls (HC); Table S1: Linear mixed-effects model evaluating longitudinal changes in body mass index (BMI), heart rate, serum creatinine, phosphorus, magnesium, and ferritin across the three study time points; Table S2: Pairwise post hoc comparisons between study time points following linear mixed-effects model analysis of BMI, heart rate, serum creatinine, phosphorus, magnesium, and ferritin; Table S3: Significant associations between genus-level gut microbiota and potential confounding variables identified by MaAsLin2 analysis at the baseline (T0). MaAsLin2 analysis was performed on genus-level relative abundances at the baseline (T0). Clinical, laboratory, and psychological variables were evaluated separately because of the limited sample size. *p*-values were adjusted for multiple testing using the Benjamini–Hochberg false discovery rate (FDR) procedure. Associations with q < 0.05 were considered statistically significant. Table S4: Linear mixed-effects model evaluating longitudinal changes in host biomarkers across the three study time points; Table S5: Pairwise post hoc comparisons between study time points following linear mixed-effects model analysis of host biomarkers.
