# Supplementary material for: Host–Microbiota Interactions in Adolescents with Severe Anorexia Nervosa: Linking Gut Dysbiosis, Intestinal Barrier Function and Microbial Metabolism
Source: Nutrients. 2026 Sep 23;18(19):3136. doi: 10.3390/nu18193136 (PMC13666687; doi:10.3390/nu18193136)
Supplement: Supplementary file 1 [file nutrients-18-03136-s001.zip › nutrients-4531734-supplementary.pdf]

Supplementary figures

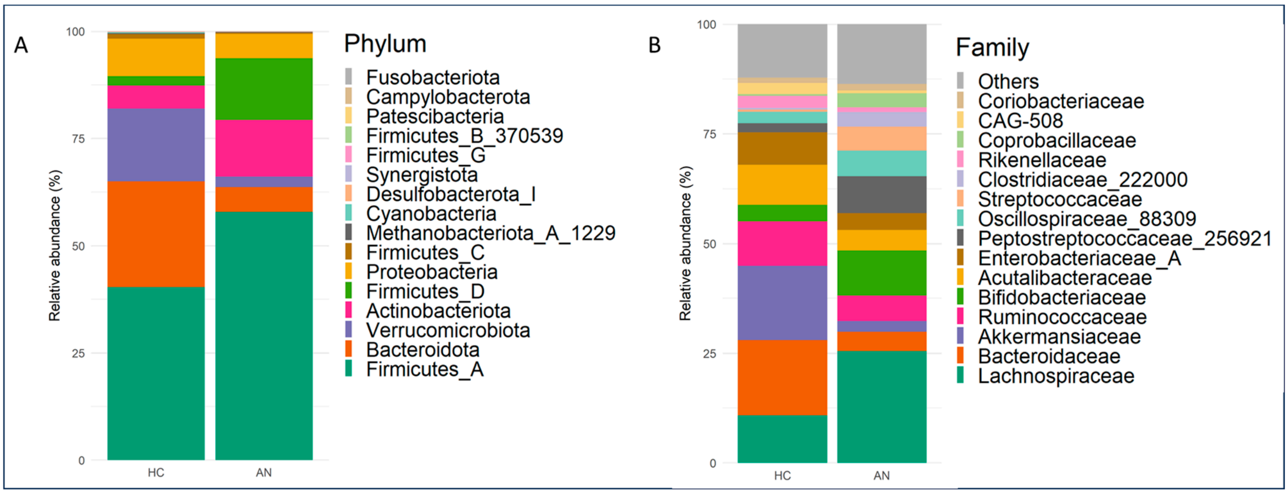

Figure S1. Relative abundance of dominant bacterial taxa in adolescents with severe anorexia nervosa (AN) at baseline (T0) and healthy controls (HC). Relative abundances of bacterial taxa are shown as stacked bar plots at the phylum (A) and family (B) levels. In panel (B), the 15 most abundant bacterial families are displayed individually, whereas all remaining families are grouped into the category "Others" (gray). Relative abundances are expressed as percentages of the total bacterial community.

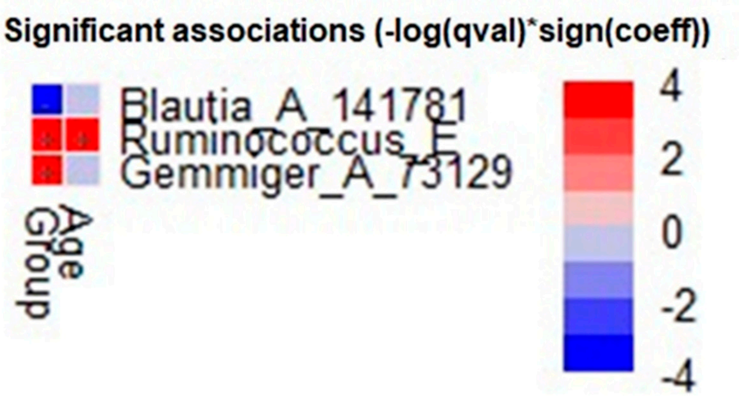

Figure S2. Multivariable association analysis of differentially abundant bacterial taxa using MaAsLin2. Associations between bacterial taxa and the metadata variables disease status (Groups) and age were evaluated using multivariable linear models implemented in MaAsLin2. Only statistically significant associations after false discovery rate (FDR) correction ($q < 0.05$) are shown. Color intensity represents the signed association strength [−log10(q-value) × sign(β)], with red indicating positive associations and blue indicating negative associations. The analysis identified *Ruminococcus*_E as the only taxon significantly associated with both disease status and age, whereas the remaining significant taxa were associated exclusively with disease status.

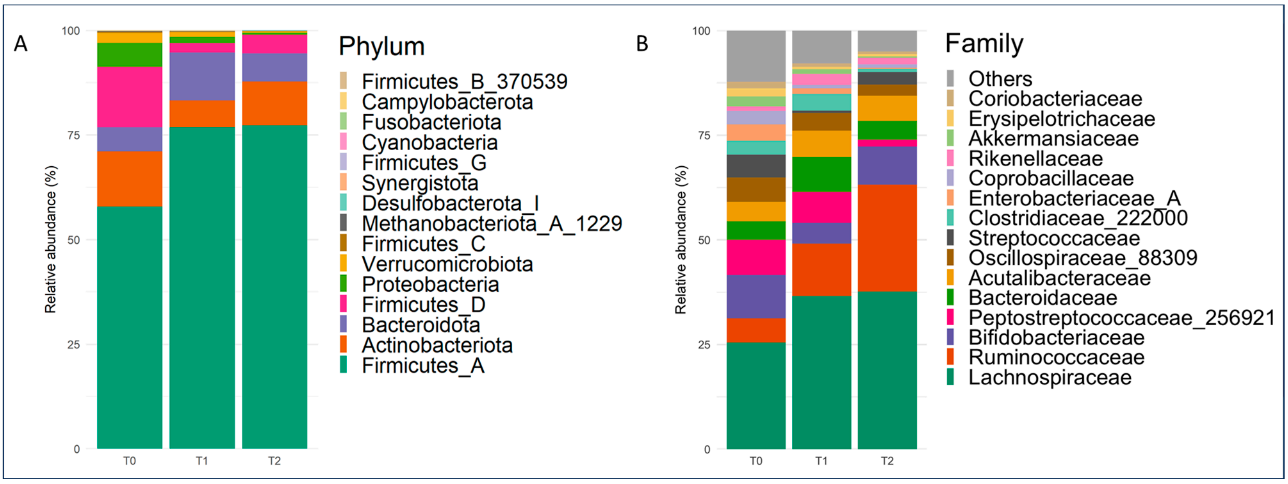

Figure S3. Relative abundance of dominant bacterial taxa across the three study time points in adolescents with severe anorexia nervosa. Relative abundances of bacterial taxa at hospital admission (T0), after one month (T1), and after three months (T2) are shown as stacked bar plots at the phylum (A) and family (B) levels. In panel (B), the 15 most abundant bacterial families are displayed individually, whereas all remaining families are grouped into the category "Others" (gray). Relative abundances are expressed as percentages of the total bacterial community.

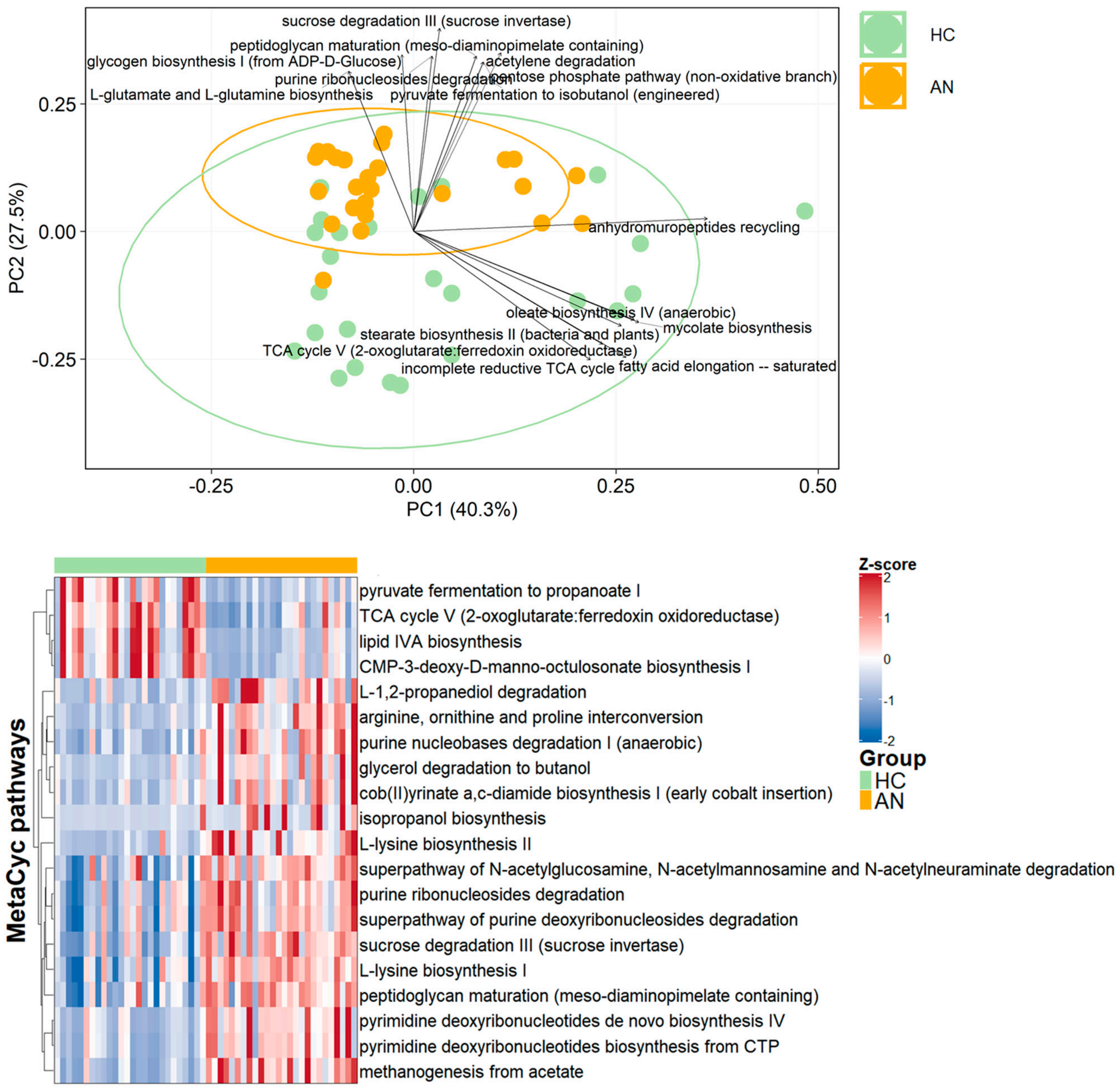

Figure S4. Predicted functional profiling of the gut microbiota in adolescents with severe anorexia nervosa (AN) at baseline (T0) and healthy controls (HC). Predicted functional profiling of the gut microbiota was inferred using PICRUSt2. (A) Principal Component Analysis (PCA) based on predicted MetaCyc pathway abundances. Each point represents an individual fecal sample, and ellipses indicate the 95% confidence interval for each group (HC, green; AN, orange). Arrows indicate the MetaCyc pathways with the highest loadings contributing to the separation along the first two principal components (PC1 = 40.3%; PC2 = 27.5%). (B) Heatmap of MetaCyc pathways significantly associated with the study group, identified using Microbiome Multivariable Association with Linear Models 2 (MaAsLin2). Age was included as a covariate in the linear model to account for its potential confounding effect, and only pathways independently associated with group status were retained. Values are displayed as row-wise Z-scores of predicted pathway abundances, with red indicating relatively higher predicted abundance and blue indicating relatively lower predicted abundance. Columns represent individual samples, whereas rows correspond to MetaCyc pathways. Hierarchical clustering was applied to pathways.

Supplementary Tables

Table S1. Linear mixed-effects model evaluating longitudinal changes in body mass index (BMI), heart rate, serum creatinine, CDI2, MASC2, phosphorus, magnesium, and ferritin across the three study time points (T0, T1, and T2). Time was included as a fixed effect and participant as a random effect. F-values, numerator (NumDF) and denominator (DenDF) degrees of freedom, raw P-values, and adjusted P-values are reported.

**LINEAR MIXED MODEL**					
**Variable**	**F_value**	**NumDF**	**DenDF**	**P_value**	**FDR**
BMI	39.74	2	48.18	0	0
Heart Rate	15.18	2	48.07	7.8E-06	7.8E-06
Creatinine	7.80	2	18.68	0.003	0.012
CDI2	7.53	2	36.31	0.002	0.004
MASC2	5.61	2	36.50	0.008	0.008
Phosphorus	14.70	2	17.11	<0.001	<0.001
Magnesium	26.24	2	17.34	<0.001	<0.001
Ferritin	38.85	2	463.56	<0.001	<0.001

Table S2. Pairwise post hoc comparisons between study time points following linear mixed-effects model analysis of body mass index (BMI), heart rate, serum creatinine, CDI2, MASC2, phosphorus, magnesium, and ferritin. Estimated mean differences (Estimate), standard errors (SE), degrees of freedom (df), and multiplicity-adjusted P-values are reported.

**Variable**	**contrast**	**estimate**	**SE**	**df**	**p.value**
BMI	T0 - T1	1.80	0.31	49.09	9.19E-07
BMI	T0 - T2	2.88	0.34	50.38	8.74E-11
BMI	T1 - T2	1.08	0.34	50.38	0.002587
Heart Rate	T0 - T1	13.35	3.35	48.21	0.000341
Heart Rate	T0 - T2	18.39	3.65	51.37	1.83E-05
Heart Rate	T1 - T2	5.05	3.65	51.37	0.172616
Creatinine	T0 − T1	0.081	0.033	18.71	0.072
CDI2	T0 - T1	5.00	1.95	40.21	0.021
CDI2	T0 - T2	7.85	2.17	40.06	0.003
CDI2	T1 - T2	2.85	2.17	40.06	0.197
MASC2	T0 - T1	4.75	2.59	39.06	0.112
MASC2	T0 - T2	9.53	2.97	39.08	0.008
MASC2	T1 - T2	4.78	2.97	39.08	0.115
Creatinine	T0 − T2	0.136	0.034	22.05	0.002
Creatinine	T1 − T2	0.055	0.030	17.57	0.219
Phosphorus	T0 − T1	-0.702	0.131	21.19	<0.001
Phosphorus	T0 − T2	-0.621	0.205	18.14	0.021
Phosphorus	T1 − T2	0.081	0.189	14.17	0.966
Magnesium	T0 − T1	0.156	0.024	17.24	<0.001
Magnesium	T0 − T2	0.229	0.040	20.34	<0.001
Magnesium	T1 − T2	0.073	0.037	14.43	0.193
Ferritin	T0 − T1	127.635	24.779	476.51	<0.001

Ferritin	T0 − T2	176.707	24.083	955.79	<0.001
Ferritin	T1 − T2	49.072	33.911	6633.03	0.381

Table S3. Significant associations between genus-level gut microbiota and potential confounding variables identified by MaAsLin2 analysis at baseline (T0). MaAsLin2 analysis was performed on genus-level relative abundances at baseline (T0). Clinical, laboratory and psychological variables were evaluated separately because of the limited sample size. P-values were adjusted for multiple testing using the Benjamini–Hochberg false discovery rate (FDR) procedure. Associations with q < 0.05 were considered statistically significant.

**Bacterial genus**	**Potential confounding variable**	**p-value**	**q-value (FDR)**
Intestinibacter	Lymphocyte count	0.00002	0.00047
*Romboutsia*_B	Lymphocyte count	0.00017	0.00208
*Escherichia*_710834	Blood urea nitrogen	0.00034	0.00809
Akkermansia	Vitamin C	0.00038	0.00918
*Alistipes*_A_871400	Blood glucose	0.00130	0.03120

Table S4. Linear mixed-effects model evaluating longitudinal changes in host biomarkers across the three study time points (T0, T1, and T2). Time was included as a fixed effect and participant as a random effect. F-values, numerator (NumDF) and denominator (DenDF) degrees of freedom, raw *p*-values, and false discovery rate (FDR)-adjusted *p*-values are reported.

**LINEAR MIXED MODEL**					
**Variable**	**F_value**	**NumDF**	**DenDF**	**P_value**	**FDR**
Nitric Oxide	11.45	2	49.45	0.000082	0.000738
Indican	9.33	2	49.72	0.000361	0.001626
Serotonin	5.74	2	49.86	0.004415	0.011555
Occludin	5.39	2	38.89	0.005868	0.011555
Short-Chain Fatty Acids	2.59	2	43.87	0.007703	0.154981
Bile Acids	1.33	2	72.00	0.26971	0.404566
Haptoglobin	0.86	2	48.72	0.428092	0.550404
IgA	0.69	2	73.00	0.506173	0.569445
Lipopolysaccharide	0.04	2	73.00	0.956639	0.956639

Table S5. Pairwise post hoc comparisons between study time points following linear mixed-effects model analysis. Estimated mean differences (Estimate), standard errors (SE), degrees of freedom (df), and multiplicity-adjusted *p*-values are reported.

**POST HOC ANALYSIS**					
**Variable**	**contrast**	**estimate**	**SE**	**df**	**p.value**
Indican	T0 - T1	13.05	9.99	48.25	0.19773
Indican	T0 - T2	33.87	10.28	49.61	0.00544
Indican	T1 - T2	20.83	10.63	52.04	0.08324
Bile Acids	T0 - T1	-20.06	10.70	48.75	0.17768
Bile Acids	T0 - T2	-17.45	10.99	50.33	0.17768
Bile Acids	T1 - T2	2.61	11.33	53.00	0.81882

Serotonin	T0 - T1	57.77	84.22	47.68	0.49611
Serotonin	T0 - T2	269.34	86.81	48.75	0.00956
Serotonin	T1 - T2	211.57	90.18	50.76	0.03438
Nitric Oxide	T0 - T1	166.42	38.69	50.30	0.000235
Nitric Oxide	T0 - T2	139.86	39.82	51.70	0.0014
Nitric Oxide	T1 - T2	-26.55	41.77	52.87	0.52767
Occludin	T0 - T1	-5.57	2.32	39.57	0.031497
Occludin	T0 - T2	4.27	3.20	42.59	0.188966
Occludin	T1 - T2	9.85	3.22	41.76	0.011576
Short-Chain Fatty Acids	T0 - T1	-0.38	2.08	45.79	0.85616
Short-Chain Fatty Acids	T0 - T2	-4.91	2.36	46.49	0.097324
Short-Chain Fatty Acids	T1 - T2	-4.54	2.40	46.20	0.097324
IgA	T0 - T1	-972.27	999.86	50.40	0.503244
IgA	T0 - T2	120.79	1067.72	54.44	0.910347
IgA	T1 - T2	1093.05	1094.08	54.03	0.503244
Lipopolysaccharide	T0 - T1	0.01	0.33	50.40	0.965677
Lipopolysaccharide	T0 - T2	0.10	0.35	54.44	0.965677
Lipopolysaccharide	T1 - T2	0.08	0.36	54.03	0.965677
Haptoglobin	T0 - T1	-10.70	8.68	50.16	0.552932
Haptoglobin	T0 - T2	-2.08	9.29	53.86	0.824105
Haptoglobin	T1 - T2	8.63	9.52	53.40	0.552932
